# The role of CAFs in therapeutic resistance in triple negative breast cancer: an emerging challenge

**DOI:** 10.3389/fmolb.2025.1568865

**Published:** 2025-03-31

**Authors:** Marianna Rita Brogna, Valeria Varone, Michele DelSesto, Gerardo Ferrara

**Affiliations:** Pathology Unit, Istituto Nazionale Tumori‐IRCCS‐Fondazione G. Pascale, Naples, Italy

**Keywords:** TNBC, CAF, therapeutic resistance, TME, cancer therapies

## Abstract

The tumor microenvironment (TME) is a crucial element of cancerous tissue and has emerged as a promising target for therapeutic strategies. The complex variety of stromal cells within the TME plays a vital role in determining the tumor’s aggressiveness and its resistance to treatment. Tumor progression is not solely driven by cancer cells harboring genetic mutations but is also significantly influenced by non-cancerous host cells within the TME, which strongly impact tumor growth, metastasis, and the response to therapies. Cancer-associated fibroblasts (CAFs) are a diverse group of stromal cells within the TME. They play dual roles, both promoting and inhibiting tumor growth, making them intriguing targets for enhancing cancer therapies. Their significant contribution to creating a tumor-supportive environment has diminished the effectiveness of various cancer treatments, including radiation, chemotherapy, immunotherapy, and hormone therapy. Research has increasingly focused on understanding how CAFs contribute to therapy resistance in triple-negative breast cancer (TNBC) to improve treatment outcomes. However, the ways in which CAF patterns affect the TME and the response to immunotherapy in TNBC are not yet well understood and the interactions between CAFs, tumor cells, and immune cells in TNBC remain largely unexplored. In this review, we thoroughly exam ine the relationship between TNBC progression and CAF patterns. We discuss the current understanding of CAF heterogeneity, their role in tumor progression, and their impact on the tumor’s response to therapeutic agents in TNBC. Additionally, we explore the potential and possible strategies for therapies targeting CAFs.

## Introduction

Breast cancer is the most common cancer among women and the second most prevalent cancer worldwide, causing approximately 600,000 deaths annually. While the overall mortality rate for breast cancer is gradually decreasing thanks to improved early detection and treatments, progress in managing advanced stages of the disease remains limited (Derakhshan and Reis-Filho, 2022). Histopathological analysis reveals that breast cancer (BC) is a heterogeneous disease with distinct subtypes that differ in metastatic potential, mortality rates, and treatment responses. These subtypes are classified based on the expression of estrogen receptor (ER), progesterone receptor (PR), and HER2. This classification defines four main biological subtypes: Luminal A (ER+/PR+/−), Luminal B (ER+/PR+/−/HER2+), HER2-positive (HER2+), and triple-negative (ER–/PR–/HER2–) (Derakhshan and Reis-Filho, 2022; Yin et al., 2020).

Triple-negative breast cancer (TNBC) represents approximately 10%–20% of invasive breast cancers. Its aggressive nature and absence of targeted treatments lead to the highest mortality rate among all breast cancer subtypes, estimated at about 20%. TNBC is characterized by a high histological grade, a high rate of positive lymph node metastasis, and a significant tendency for recurrence and distant metastasis compared to other breast cancer types (Yin et al., 2020).

TNBC exhibits positive GPER(G protein-coupled estrogen receptor), but negative amplification/overexpression of human epidermal growth factor receptor 2 (HER2), progesterone receptors (PR), and estrogen receptors (ER), therefore reducing the options of targeted therapies. The treatment of triple-negative breast cancer (TNBC) is difficult and often viewed as a “black hole” compared to other breast cancer subtypes, due to chemo-resistance and poor prognosis (Yin et al., 2020; Li et al., 2022). TNBC outcomes are generally unfavorable because of the disease’s heterogeneity and the absence of specific therapeutic targets. Systemic chemotherapy remains the primary adjuvant treatment for TNBC, with anthracycline and taxane-based regimens being commonly utilized due to their considerable effectiveness in treating this subtype. Thus, identifying molecular biological factors that can influence or predict the prognosis of triple-negative breast cancer (TNBC) and investigating their mechanisms is crucial for understanding the onset and progression of TNBC(3). The behavior of breast cancer is significantly and intricately influenced by the tumor microenvironment. Triple-negative breast cancer (TNBC) has a distinct microenvironment compared to other breast cancer subtypes. This unique surrounding inhibits apoptosis and the immune response against the tumor, encourages angiogenesis, increases drug resistance, and promotes cancer cell proliferation (Derakhshan and Reis-Filho, 2022; Yin et al., 2020; Li et al., 2022). In this review, we analyze in depth the connection between the progression of triple-negative breast cancer (TNBC) and the characteristics of cancer-associated fibroblasts (CAFs). We highlight the current knowledge regarding the heterogeneity of CAFs, their contribution to tumor development, and their influence on how TNBC responds to therapeutic treatments. Furthermore, we examine the potential of therapies aimed at targeting CAFs and the strategies that could be employed to implement such treatments.

## Impact of tumor microenvironment in TNBC

**FIGURE 1:**
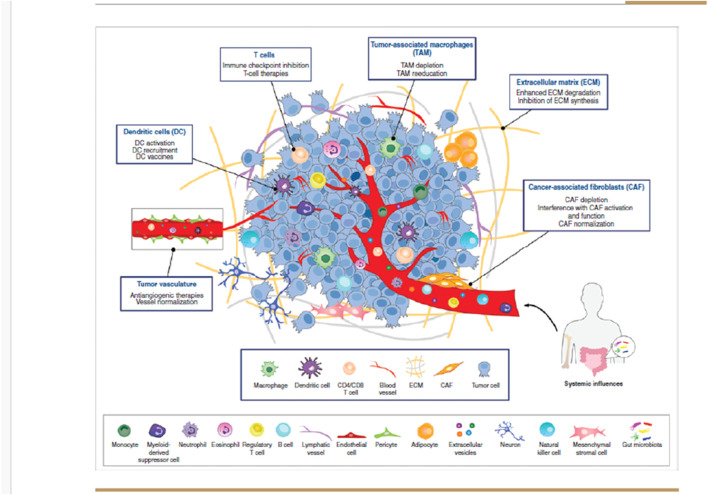
Composition of the tumour microenvironment. The tumor microenvironment (TME) consists of a variety of cell types and secreted factors that serve as potential targets for anticancer therapies. It includes heterogeneous cancer cells, immune cells such as T and B lymphocytes, tumor-associated macrophages (TAMs), dendritic cells (DCs), natural killer (NK) cells, myeloid-derived suppressor cells (MDSCs), neutrophils, and eosinophils. Additionally, stromal cells like cancer-associated fibroblasts (CAFs), pericytes, and mesenchymal stromal cells, along with blood and lymphatic vascular networks, as well as tissue-specific cells such as neurons and adipocytes, are also present. These cells release extracellular matrix (ECM) components, growth factors, cytokines, and extracellular vesicles (EVs), which play a crucial role in cell communication within the TME and beyond. Since each cell type uniquely influences tumor progression and therapeutic response, multiple TME-targeted therapies have been developed. The most advanced approaches, either approved or in clinical trials, primarily focus on TAMs, DCs, T cells, tumor vasculature, ECM, and CAFs.

The tumor microenvironment (TME), which includes cancer cells, stromal cells, infiltrating immune cells, and other supportive elements, plays a pivotal role in regulating tumor cell growth, invasion, and migration. This environment promotes tumor initiation and progression through the production of growth factors, hormones, and cytokines. Numerous studies have underscored the importance of the TME as the “soil” that supports the “seed” (cancer cells). Tumor metastasis primarily occurs through a dynamic interaction between tumor cells and host tissue, involving processes such as adhesion, proteolysis, invasion, and angiogenesis (Xiao and Yu, 2021) (Figure 1).

In the case of the aggressive triple-negative breast cancer (TNBC) subtype, the TME is a critical driver of disease progression. The TNBC microenvironment, composed of an altered extracellular matrix, soluble mediators, immune cells, and reprogrammed fibroblasts, undermines the host’s antitumor defenses while promoting tumor growth and metastasis. A key challenge in TNBC is the tumor stroma, which surrounds blood vessels and acts as a major obstacle to the effective delivery of drugs from the bloodstream to tumor tissues. Furthermore, the presence of dense stromal deposits and the accumulation of stromal cells significantly influence where and how therapeutic agents penetrate the tumor (Hu et al., 2022; Deepak et al., 2020).

In triple-negative breast cancer (TNBC), the complexity of the stroma is not well understood, thus limiting the stromal cell-targeted therapies. Hence, it is crucial to explore how the interactions among the tumor, stroma, and inflammation impact features that facilitate metastasis in TNBC.

## The origins of CAFs in breast cancer

Cancer-associated fibroblasts (CAFs) are activated fibroblasts within the tumor microenvironment that play a crucial role in supporting tumor cell growth and survival. They perform functions such as: (i) releasing soluble factors, including growth factors like VEGF, PDGF, FGF, and cytokines, which stimulate tumor cell proliferation; (ii) altering the tumor microenvironment by remodeling the extracellular matrix or changing its pH; and (iii) promoting angiogenesis, which improves tumor metabolism, supports cell proliferation, and facilitates metastasis by enabling the release of circulating tumor cells. Moreover, CAFs contribute to tumor cell resistance to chemotherapy, making them a key focus in the development of targeted cancer therapies (Öhlund et al., 2014).

### Origin of CAFs

The origin of each CAF type determines its unique characteristics, which in turn affect its functional role within the tumor microenvironment. Despite numerous proposed markers for CAFs, none are fully specific or universally accepted, making it difficult to trace their origins. CAFs are believed to primarily arise from activated fibroblasts, with alternative sources including adipocytes and mesenchymal stem cells (MSCs) (Öhlund et al., 2014; Yang et al., 2023).

### Local resident fibroblasts and transition from NFS(NORMAL fibroblast) to CAFs

**FIGURE 2:**
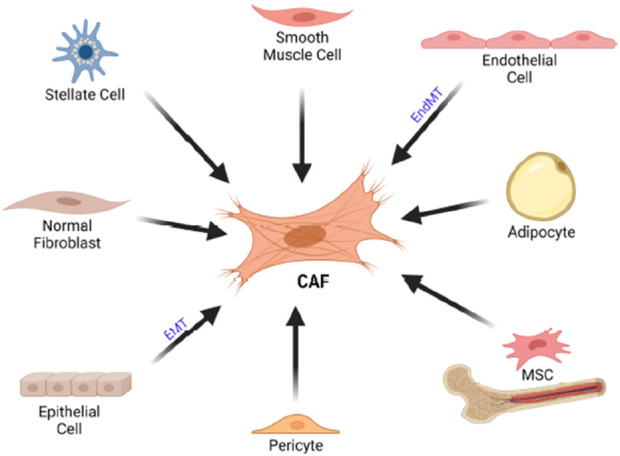
Cellular origin of CAF. CAFs can originate from various cell typoe such as stellate cells, endothelial cells, adiphocyte,MSCs, pericytes, epithelial cells as well as normal fibroblast. CAF, cancer associated fibroblast. EMT, epitheliual mesenchimal transition, EndMT, endothelial to mesenchimal transition; MSC, mesenchimal stem cells.

Most CAFs are believed to originate from the activation of resident fibroblasts in local tissues. These fibroblasts, which produce the extracellular matrix (ECM), become activated in response to tissue damage, producing TGF-β and acquiring a contractile phenotype with increased α-SMA levels, thereby forming myofibroblasts. The main distinction between this process and active inflammation is that the fibroblasts in cancer do not revert to their inactive state. This is why tumors are often described as “wounds that do not heal (Buyukhatipoglu et al., 2015). Figure 2.

#### Differences between NFs and CAFs

Cancer-associated fibroblasts (CAFs) display more aggressive phenotype than normal fibroblasts (NFs), such as increased proliferation, migration, invasion, tumorigenicity, and chemoresistance. Studies identifying differentially expressed genes in CAFs revealed upregulation of genes involved in signaling, ECM remodeling, and cell migration, while downregulating genes related to steroid hormone metabolism and detoxification. A 2012 study analyzing miRNA expression in normal fibroblasts (NFs) and cancer-associated fibroblasts (CAFs) from breast cancer patients found that miR-221-5p, miR-31-3p, and miR-221-3p were upregulated in CAFs, while miR-205, miR-200b, and others were downregulated. These miRNAs regulate key processes such as proliferation, adhesion, and migration, reinforcing the tumor-promoting function of CAFs. In 2013, gene expression analysis identified 809 genes upregulated in CAFs, including CCL18, CXCL12, MMP9, and PLK1, which are involved in cell cycle regulation, adhesion, and secretion, further supporting breast cancer progression (Gunaydin, 2021).

## Bone marrow-derived fibroblasts

Bone marrow-derived fibroblasts (BM-DFs) can convert into CAFs in breast cancer, promoting tumor growth and angiogenesis. Their recruitment decreases PDGFRα-positive CAFs, which is associated with poorer prognosis and highlights their impact on tumor progression.

## Mesenchymal stem cells

Bone marrow-derived mesenchymal stem cells (MSCs) can transform into CAFs and enhance breast cancer metastasis via CCL5 paracrine signaling. TGF-β from cancer cells induces adipose tissue-derived stem cells to differentiate into CAFs through the SMAD3 pathway. Additionally, exosomes from breast cancer cells activate SMAD signaling to trigger this transformation, upregulating markers like α-SMA and TGF-β(11).

### Adipocytes

Wnt3a secreted by tumor cells can convert adipocytes into CAFs in breast cancer by activating the Wnt/β-catenin pathway, leading to increased FSP-1 expression, but not α-SMA.

### Pericytes

In 2016, it was found that pericytes could be converted into fibroblasts through the activation of PDGF-BB-PDGFRβ signaling, which promoted thyroid cancer invasion and metastasis. This finding was later supported by evidence from a breast cancer model, where a subgroup of CAFs expressing vascular-regulating genes and located near the vasculature was identified. It was concluded that this subgroup of CAFs originated from perivascular cells (Folgueira et al., 2013; Venning et al., 2021).

### CAF subtypes

Studies using mouse models of breast cancer have identified distinct CAF subpopulations, each with specialized roles in tumor progression. Single-cell RNA sequencing has revealed that these subpopulations have unique transcriptional profiles that evolve over time and with tumor stages. The dynamic composition of CAFs influences clinical outcomes, particularly in triple-negative breast cancers (TNBC) with BRCA mutations, where the balance between key CAF subtypes significantly impacts disease progression and treatment response (Sarkar et al., 2023; Li Q. et al., 2021).

CAF subtypes can be broadly categorized into three groups:

1. Quiescent CAFs (qCAFs)
2. Tumor-restraining CAFs (rCAFs)
3. Tumor-promoting CAFs (pCAFs)

qCAFs and rCAFs are more common in early-stage cancers, while pCAFs dominate in advanced stages, highlighting the dynamic role of CAFs in tumor progression. CAFs in breast cancer can be classified into subtypes (CAF-S1 to CAF-S4) based on specific markers like CD29, FAP, FSP1, α-SMA, CAV1, and DPP4(14,15).

CAFs-I: These fibroblasts are involved in cell adhesion and extracellular matrix remodeling. They are also associated with a poor response to immunotherapy.

CAFs-II: Characterized as inflammatory CAFs(iCAFs), this subset plays a key role in inflammation within the tumor microenvironment.

CAFs-III: These fibroblasts exhibit features typical of myofibroblasts, such as the expression of alpha-smooth muscle actin (α-SMA), and are involved in tissue remodeling and fibrosis.

CAFs-IV: This subgroup is involved in antigen presentation, potentially influencing immune responses in the tumor microenvironment.

Inflammatory CAFs are closely linked to triple-negative breast cancer (TNBC), but their marker genes lack specificity, complicating their identification in the stroma. CAF subpopulations can have contrasting effects: CD146-positive CAFs support ER expression and tamoxifen sensitivity in ER-positive breast cancer, while CD146-negative CAFs contribute to tamoxifen resistance, highlighting the complexity and context-dependent roles of CAFs in cancer progression and therapy response (Wang et al., 2023; Costa et al., 2018; Liu et al., 2022). In more recent studies, CAFs have been further subdivided into various subsets based on specific markers and functions, including:

- FAPα-type CAFs (fibroblast activation protein alpha),
- FSP1-type CAFs (fibroblast-specific protein 1),
- PDGFRα-type CAFs (platelet-derived growth factor receptor alpha),
- PDGFRβ-type CAFs (platelet-derived growth factor receptor beta).

### Biomarkers of CAFs

CAFs can be identified and quantified using various markers, which serve as diagnostic and prognostic tools, often indicating a negative outcome. CAFs differ from normal fibroblasts (NFs) in behavior, function, and protein expression, making these proteins potential biomarkers for differentiation. However, the heterogeneity of CAFs limits the specificity and sensitivity of these markers (Lee et al., 2018).

### α-SMA

α-SMA is a skeletal protein used as a marker for activated fibroblasts and is linked to TGF-β production and a contractile phenotype. While α-SMA-positive CAFs are involved in cancer progression, immune suppression, and therapy resistance, their role remains controversial. In some models, removing α-SMA-positive CAFs accelerated cancer growth, while in others, higher expression correlated with better prognosis. In breast cancer, α-SMA-positive myofibroblasts are associated with tumor cell proliferation and poor survival outcomes, promoting tumor progression by supplying nutrients like lactate and pyruvate to cancer cells (Kahounová et al., 2018).

## Vimentin

Vimentin, a type III intermediate filament protein, is a marker of cellular structure and motility, often expressed at high levels in CAFs and linked to migration and invasion. Its increased expression in the stromal compartment is associated with higher tumor malignancy and shorter survival in colorectal cancer and pancreatic ductal adenocarcinoma. Vimentin is also expressed by normal fibroblasts, mesenchymal cells (adipocytes, myocytes), and epithelial cells undergoing the EMT (Lee et al., 2018; Kahounová et al., 2018).

### FSP1 (fibroblast-specific protein 1)

FSP1 (S100A4) is a common CAF marker, with its positive expression linked to lymphovascular invasion and metastasis in cancers like colorectal and urothelial carcinoma. However, FSP1-positive fibroblasts may also enhance immune surveillance by producing collagen and capturing carcinogens. In breast cancer, FSP1-positive CAFs promote metastasis by secreting VEGF-A and tenascin-C, with higher expression in ILC than NST. A high ratio of FSP1-positive CAFs is associated with prolonged RFS and OS, and FSP1 is also expressed in breast cancer cells, particularly in breast cancer inflammatory subtype.

### FAP (fibroblast activation protein)

FAP is a key biomarker of CAFs involved in ECM remodeling and fibrosis, promoting tumor progression and creating an immunosuppressive TME. High FAP expression in CAFs is linked to poor prognosis in ovarian cancer. Despite limited success in clinical trials, FAP-targeted therapies, including gene knockout, small molecules, and immunotherapies, remain promising and require further investigation. In breast cancer, FAP-positive CAFs mediated Treg activation and exerted immunosuppressive activity in a dipeptidyl peptidase (DPP) 4-dependent manner that was related to a poor outcome. Unexpectedly, a study also indicated a positive relationship between abundant FAP expression and longer OS and DFS of patients with IDC (Kim et al., 2015; Røgenes et al., 2024).

### PDGFRα and PDGFRβ

PDGFRα/β-positive CAFs promote macrophage migration and M2 polarization, influencing the immune microenvironment. Inhibiting PDGFR signaling transforms CAFs into resting fibroblasts, suppressing angiogenesis and tumor growth, making PDGFR-targeted therapies a potential treatment strategy. PDGFRα and PDGFRβ are widely expressed in fibroblasts and various cancer cells, with PDGFRβ expression in stromal cells in breast cancer correlating positively with histopathological grade and HER2 expression, but negatively with ER expression. PDGFRβ levels in stromal cells also correlate with reduced tamoxifen efficacy, poorer radiotherapy response, and worse prognosis, particularly in young and premenopausal patients (Røgenes et al., 2024).

## Caveolin

Caveolins (Cavs), including Cav-1, Cav-2, and Cav-3, are key proteins in caveolae membranes. The role of Cav-1 in fibroblast phenotypes is controversial: Cav-1 depletion in NIH-3T3 fibroblasts promotes cell growth, while its expression enhances the release of inflammatory and tumor-promoting factors, supporting tumor cell proliferation and migration. Cav-1 is also crucial for fibroblast-mediated microenvironmental remodelling (Røgenes et al., 2024; Yamamoto et al., 2023).

In breast cancer, Cav-1 expression is downregulated in CAFs and correlates positively with patient prognosis, though some studies suggest the opposite. Cav-1 knockout reduces fibroblast contractility, and higher Cav-1 expression in CAFs from metastatic lymph nodes indicates a role in metastasis. Cav-1 is also linked to the “reverse Warburg effect” in CAFs, promoting cancer malignancy.

### PDPN (podoplanin)

Studies show that PDPN expression in CAFs predicts poor prognosis in various cancers, including lung, breast, and pancreatic cancer, and is linked to increased tumor progression Invasive breast cancer with PDPN-positive CAFs tends to have a more aggressive pathological status, characterized by higher lesion-to-muscle ratios and rim enhancement in imaging. PDPN promotes fibroblast migration and accelerates endothelial cell pseudotube formation, contributing to breast cancer development and metastasis.

### ASPN

Asporin (ASPN), a small leucine-rich proteoglycan expressed predominantly by cancer associated fibroblasts (CAFs), plays a pivotal role in tumor progression. ASPN promotes activation of p-EGFR and its effector p-ERK1/2. It has been also observed that ASPN secreted from CAFs activates Rac1 via interaction with CD44, thereby promoting invasion by CAFs (Yamamoto et al., 2023).

### STC1 (stanniocalcina)

ANGPTL4(Angiopoietin-related 4 Protein), MMP13 (matrix metalloproteinase 13,collagenase 3) and STC1 are identified as STAT3-dependent mediators of CAF pro-tumorigenic functions. STC1, a secretory glycoprotein, plays an oncogenic role in TNBC, promoting invasion, metastasis, and potentially chemotherapy resistance. A retrospective study linked STC1 expression in breast cancer to chemotherapy resistance, though the precise molecular mechanisms remain unclear. STC1 is induced by hypoxia, with HIF-1α binding to its promoter, suggesting its role in resistance. Further research is needed to validate and detail these mechanisms (Friedman et al., 2020). Table 1.

**TABLE 1:** Biomarkers of CAFs.

actin (α-SMA) of smooth muscle	Marker of myofibroblasts
Fibroblast activation protein (FAP)	Marker of myofibroblasts
Tenascina-C	Regulates tumor cell adhesion during invasion
Periostina	Produced as a result of the tissue repair process
Neuron glial antigen-2 (NG2)	Associated with pericytes, which can sometimes give rise to fibroblasts
Vimentina	Protein associated with the plasma membrane
Desmina	Marker of blood vessel maturation (manifestation of angiogenesis
Platelet derived growth factor receptor-α e β (PDGFR α e β)	Tyrosinchinase receptor upregolated in CAFs
Fibroblast specific protein-1 (FSP-1)- S100A4	Marker of myofibroblasts
ASPN	New potential marker of CAFs

## Value of CAFs for breast cancer diagnosis and prognosis prediction

CAFs play a vital role in breast cancer progression and prognosis, driving research into their potential for early detection and prognostic prediction. Elevated levels of type IX and X collagen α1 and cartilage ligament matrix were found in the plasma of breast cancer patients, compared to those with benign lesions or healthy controls. *In vitro*, these proteins’ expression increased in fibroblasts exposed to tumor cell-conditioned medium. This suggests that fibroblast expression of certain proteins could serve as biomarkers to distinguish malignant from benign tumors. The high, specific expression of FAP in tumors makes it a promising target for both imaging and therapy, with Loktev et al. developing a radiotracer based on a FAP-specific enzyme inhibitor (FAPI).

### Secreting CAFs’ factors

In breast cancer, CAFs enhance proliferation, migration, invasion, and stemness of tumor cells, influencing to treatment resistance, and remodel the ECM. They also regulate cancer cell metabolism and suppress immune functions, further supporting tumor growth.

CAFs-derived TGF-β drives EMT and enhances fibronectin, vimentin, MMP-2/9, SNAIL, and TWIST expression, promoting breast cancer cell motility, drug resistance, and stemness via upregulation of lncRNA HOTAIR, silencing tumor suppressor genes.

CAFs secrete HGF, enhancing breast tumorigenesis and colony formation, suppressing the luminal phenotype and sustaining the triple-negative status of breast cancer cells. In addition, CAFs-secrete FGF5 supporting a chemoresistant CSC phenotype and activates HER2 via the FGFR2/c-Src/HER2 axis, leading to resistance to HER2-targeted therapies (Xie et al., 2022).

#### Interleukins

CAFs-derived IL-6 enhances breast cancer cell invasion and progression from *in situ* to invasive cancer, while promoting resistance to apoptosis in luminal cancer cells. IL-6 and IL-8 from chemoresistant CAFs enrich CSCs by creating niches, and IL-32 in CAFs binds integrin β3, activating p38 MAPK signaling, upregulating EMT markers, and promoting tumor invasion.

#### Chemokines

CAFs exposed to neoadjuvant chemotherapy secrete chemokines like Glu-Leu-Arg (ELR) motif-positive chemokines, enhancing breast cancer cell invasion through CXCR2. Activated by immunosuppressive S100A9-positive myeloid cells, CAFs secrete CCL16, forming a feedback loop that switches the stroma to a reactive mode. CAF-derived CXCL12 promotes cancer cell growth, invasion, and stemness by altering cell motility and increasing vascular permeability. CXCL12 facilitates tumor invasion and metastasis, while CCL11, CXCL14, CCL6, and CCL12 further contribute to chemoresistance and metastasis by enhancing glycolysis (Galbo et al., 2021).

#### Other proteins

CAFs secrete Tenascin-C, which reduces breast cancer cell apoptosis and aids in metastatic foci formation. CAF-derived osteopontin, gremlin1, promote migration, invasion, and EMT in triple negative breast cancer cells. Neurotrophins, released by CAFs, are implicated in tumor cell proliferation, metastasis, resistance, angiogenesis, and CSC self-renewal. Although not widely studied in breast cancer, neurotrophins released by CAFs are believed to contribute to tumor malignancy and represent potential therapeutic targets (Galbo et al., 2021; Qin et al., 2023).

#### Exosomes

Exosomes from breast cancer cells play a key role in transforming normal fibroblasts (NFs) into cancer-associated fibroblasts (CAFs) by transferring specific miRNAs and proteins. miRNAs like miR-9, miR-125b, and miR-222 promote CAF-like traits, enhancing tumor stemness, migration, and invasion. Protein-containing exosomes, such as survivin, activate fibroblasts by increasing SOD1 expression.

This transformation supports breast cancer progression, with NFs also contributing to an IL-1β-enriched microenvironment, potentially promoting tumor recurrence in surrounding (Yang et al., 2019).

✓ Exosomal DNA In 2017, it was reported that CAF-derived exosomes containing mitochondrial DNA (mtDNA) counteracted oxidative phosphorylation deficiencies in breast cancer, contributing to hormone therapy resistance.
✓ Exosomal non-coding RNAs Exosomal miRNAs from CAFs play a crucial role in breast cancer cell communication. CAF-derived miR-221 and miR-222 reduce ER expression in ER-negative cancers, while miR-22 targets ERα and PTEN, promoting tamoxifene resistance and stem cell formation.
✓ Exosomal miRNAs (miR-21, miR-378e, miR-143, miR-181d-5p, miR-3613-3p, miR-500a-5p, miR-18b, and miR-1-3p) from CAFs promote EMT, stemness, proliferation, metastasis, and invasion of breast cancer cells by targeting various genes, including CDX2, HOXA5, SOCS2, USP28, and GLIS1. Downregulation of miRNAs in CAF-derived exosomes promotes breast cancer malignancy, with loss of miR-7641 enhancing stemness and glycolysis by upregulating HIF-1α in cancer cells.
✓ Non-coding RNAs from CAFs contribute to breast cancer malignancy by promoting therapy resistance and metastasis. CAF-derived exosomal RNA activates STAT1-dependent signaling, while RN7SL1 and SNHG3 in exosomes enhance growth, metastasis, and metabolic shifts in cancer cells, fostering resistance and altered metabolism. While pre-clinical studies show promise, few have explored gene-editing of non-coding RNAs in CAFs for cancer treatment. Although these therapeutic strategies show potential for clinical use, they face several challenges, including difficulties in delivery, the short half-life of RNA molecules, activation of the innate immune response, and off-target effects.
✓ Exosomal proteins CD81-positive exosomes from CAFs enhance breast cancer cell motility by activating Wnt-PCP signaling and promoting metastasis through the Wnt/β-catenin pathway. CAF-derived exosomes also contain metalloproteinase ADAM10, which activates RhoA and upregulates ALDH, promoting migration. Additionally, hypoxic CAF-derived exosomes increase MMP-9 and IL-8 expression, enhancing cancer cell invasion via GPR64, with BNIP3 phosphorylation regulating the process (Li H. et al., 2021; Zou et al., 2024).

#### Releasing nutrients

The “reverse Warburg effect” refers to glycolysis in CAFs that produces lactate and pyruvate, which support energy generation in cancer cells, promoting cancer progression. Studies have shown that autophagy-mediated loss of Cav-1 in CAFs leads to mitochondrial dysfunction and oxidative stress, resulting in the release of nutrients and substrates that fuel breast cancer cell growth, stemness, and proliferation.

Tumor cells reprogram CAFs into an aerobic glycolysis model through the estrogen GPER/cAMP/PKA/CREB pathway, enhancing mitochondrial activity and conferring treatment resistance (Singer et al., 2008).

Exosomal miR-105 from MYC-expressing cancer cells reprograms CAF metabolism, fueling nearby cancer cell growth. Additionally, hypoxia increases glycolysis in CAFs by activating ATM-mediated phosphorylation of GLUT1 and upregulating pyruvate kinase M (PKM). Metabolically reprogrammed CAFs release lactate into the microenvironment, promoting breast cancer cell invasion through the TGF-β1/p38 MAPK/MMP2/-9 signaling axis and enhancing mitochondrial activity. Epigenetic changes dysregulate HIF-1α and metabolic enzymes like fructose1,6-bisphosphatase FBP1, PKM, and Lactate dehydrogenase A (LDHA) in CAFs, fueling cancer growth. HMGB1 (high mobility group box-1 protein) from breast cancer cells also triggers aerobic glycolysis in CAFs, further promoting metastasis.

#### Growth factors, cytokines and other ligands

In 2010, Kojima et al. showed that TGFβ and CXCL12 autocrine signaling could transform normal fibroblasts into CAFs. Similarly, Zhang et al. identified IDH3α as a key regulator of fibroblast metabolic reprogramming, promoting CAF transformation via TGF-β and PDGF. TGFβ and CXCL12 autocrine signaling, along with IDH3α-regulated metabolic reprogramming, promote the transformation of normal fibroblasts into CAFs.

Osteopontin, secreted by breast cancer cells, reprograms fibroblasts into a proinflammatory state, inducing Epithelial Mesenchimal Transition (EMT) in cancer cells via CXCL12 secretion (Singer et al., 2008; Liubomirski et al., 2019; Nurmik et al., 2020).

## Role of CAF in triple negative breast cancer

Cancer-associated fibroblasts (CAFs) are specialized fibroblasts found surrounding the tumor, and they play a crucial role in the tumor microenvironment. Research has shown that CAFs have increased proliferation, migration, and collagen secretion abilities compared to normal fibroblasts (NFs), and they can promote the migration and invasion of breast cancer cells. While normal fibroblasts help prevent tumor formation, CAFs enhance several tumor characteristics, including cancer cell proliferation and invasion, neo-angiogenesis, inflammation, and remodeling of the extracellular matrix (ECM). CAFs are also known to suppress anti-tumor immunity and contribute to resistance to therapy. The direct impact of CAFs on the therapeutic response, potentially leading to therapy resistance, helps explain their connection to poor outcomes. One common hypothesis is that the ECM modified by CAFs alters the physical properties of the tissue, affecting drug permeability and, in turn, the effectiveness of treatment (Liubomirski et al., 2019).

CAFs play a key role in synthesizing and reshaping the ECM through the “desmoplastic reaction,” producing collagen types I, III, IV, and V, fibrinolytic protein, hyaluronic acid, and laminin while degrading the ECM via proteases like MMPs and urokinase-type plasminogen activator. This process hardens tissues, promotes stromal fibrosis, and remodels the TME, creating a scaffold that hinders immune cell and drug penetration, leading to immune evasion and drug resistance. Additionally, the remodeled ECM fosters interactions between tumor cells and cytokines, enhancing cancer cell migration, invasion, and malignancy, contributing to cancer progression and poor patient prognosis (Nagel et al., 2022).

The actions of cancer-associated fibroblasts (CAFs) on tumor cells in triple-negative breast cancer (TNBC) contribute to tumor growth through multiple mechanisms:

1. Indirect Mechanisms: CAFs support tumor progression by promoting immune evasion,.
2. Direct Mechanisms: They activate signaling pathways within tumor cells, enhancing tumorigenesis by disrupting normal cellular functions such as cell cycle regulation and programmed cell death.

## Prognostic marker

High fat density is linked to worse prognosis and serves as a diagnostic marker for early tumor detection. Breast cancer cells reprogram fibroblasts into proinflammatory cancer-associated fibroblasts (CAFs) via osteopontin, promoting inflammation, tumor growth, and metastasis. Proteomic studies identify osteopontin’s role, while podoplanin in CAFs serves as a negative prognostic marker, though its role in breast cancer needs further study.”

CAFs influence nearby cells to adopt pro-neoplastic behaviors and have selective effects on specific tumor cells. In breast cancer, they secrete cytokines that enhance androgen synthesis, stimulate growth factors like FGF and HGF, and drive excessive epithelial cell proliferation. CAFs also induce epithelial-mesenchymal transitions (EMT) and remodel the extracellular matrix, facilitating tumor progression. One specific factor released by CAFs is fibroblast-specific protein 1 (FSP1), which alters the tumor microenvironment (TME) to promote growth. Furthermore, some CAFs can recycle waste products from anaerobic metabolism, redirecting them into alternative metabolic pathways that support tumor cell proliferation (Nagel et al., 2022; Chen et al., 2021).

### Promoting angiogenesis

CAFs promote angiogenesis in breast cancer through both VEGF-dependent and VEGF-independent mechanisms. VEGF, primarily produced by CAFs, enhances angiogenesis, particularly under hypoxic conditions via the HIF-1α/GPER pathway. IL-6 can also induce VEGF-A secretion from CAFs. Independently of VEGF, CAFs secrete CXCL12 and clusterin to stimulate angiogenesis, while FOSL2 promotes angiogenesis by upregulating Wnt5a. Additionally, the mechanical force exerted by CAFs contributes to vascular formation in the tumor microenvironment (Chen et al., 2021).

### Metastases

Cancer-associated fibroblasts (CAFs) play a significant role in promoting tumor metastasis through various mechanisms:

1. Gene Expression Modulation: CAFs can alter gene expression in tumor cells, leading to the overproduction of proteins such as heat shock factor 1 (HSF1), which are integral to cellular signaling pathways that drive metastasis.
2. Distruption of Tumor Suppressor Functions: By interfering with cell cycle regulation, CAFs can suppress the activity of tumor suppressor genes, including TP53, resulting in unchecked cellular proliferation and tumor progression.
3. Degradation of Structural Barriers: CAFs secrete matrix metalloproteinases (MMPs), enzymes that degrade the basement membrane and extracellular matrix (ECM), compromising tissue integrity and enabling cancer cells to migrate from the primary tumor site.
4. Paracrine Signaling: CAFs facilitate cancer proliferation, invasion, and metastasis by releasing signaling molecules such as vascular endothelial growth factor (VEGF), fibroblast growth factor 2 (FGF2), transforming growth factor-beta (TGFβ), CXCL12, and interleukin-6 (IL6).
5. Extracellular Matrix Remodeling: Indirectly, CAFs contribute to metastasis by altering the ECM, creating a more permissive environment for tumor cell migration and invasion.

These combined actions enable CAFs to support tumor dissemination and the establishment of metastatic sites (Lin et al., 2024; Nurmik et al., 2020).

### Chemoresistence

The main categories of overall chemoresistance induced by CAFs include ECM remodeling, paracrine signaling, induction of stem-like properties in cancer cells, metabolic manipulation, modulation of the immune environment in the TME, and exosomal shuttling between tumor cells and CAFs. CAF-induced chemotherapy resistance involves multifaceted processes that generate a physical barrier by modifying the extracellular matrix (ECM). This barrier reduces drug accessibility to tumor cells, activates pro-survival signaling pathways, and inhibits apoptotic signaling pathways. ECM remodeling by CAFs may also result in increased EMT and stem-like properties, aggressive cancer cell transitions, epigenetic modulation, and general modulation of the crosstalk between breast tumor cells and stromal components in the TME (Zheng et al., 2022). CAFs secrete type 1 collagen, which inhibits chemotherapeutic drug absorption in solid tumors.

Cancer-associated fibroblasts (CAFs) have been implicated in contributing to chemoresistance through various mechanisms:

1. Competition with Chemotherapy: CAFs can reduce the effectiveness of anticancer drugs by releasing growth factors or cytokines, or by inducing neighboring cells to produce these substances, thereby promoting tumor cell survival and diminishing drug efficacy.
2. Neutralization of Drug Effects: CAFs may secrete antiapoptotic agents or modify the tumor microenvironment, such as altering the pH, to counteract the impact of chemotherapeutic agents.
3. Enhanced Adhesion and Resistance: Tumor cells may develop resistance when drugs affect their ability to adhere to stromal cells or the extracellular matrix (ECM). For instance, TGF-beta secretion by CAFs promotes chemoresistance by strengthening tumor cell attachment to the ECM.
4. Association with Aggressive Tumors: In human breast tumors, an abundance of stromal myofibroblasts, identified by alpha-smooth muscle actin (aSMA)-positive fibroblasts, is linked to aggressive adenocarcinomas and serves as a predictor for disease recurrence.

These findings highlight the multifaceted role of CAFs in fostering a tumor-supportive environment and in contributing to resistance to both chemotherapy and immunotherapy (Zheng et al., 2022; Brennen et al., 2012).

### Reshaping the ECM and providing “mechanical pressure

Type I collagen, a key component of the breast cancer tumor microenvironment (TME), plays a crucial role in supporting cancer cell survival and aggressiveness. Cancer cells release Wnt3a, which activates the Wnt/β-catenin signaling pathway in cancer-associated fibroblasts (CAFs). This activation increases the secretion of fibronectin and type I collagen, which in turn promotes the release of MMP-9, aiding cancer cell migration. Fibronectin, secreted by CAFs in response to TGF-β and IFN-γ, stimulates several processes in breast cancer cells, including proliferation, migration, epithelial-mesenchymal transition (EMT), and angiogenesis. High fibronectin expression in breast cancer is associated with shorter patient survival and EMT promotion via the STAT3 pathway. Inhibiting fibronectin production reduces cancer cell aggressiveness. Furthermore, increased tumor stiffness, mediated by miR-18a-induced PTEN reduction, contributes to enhanced cancer cell survival, migration, and invasion.

Increased collagen crosslinking by lysyl oxidase (LOX) hardens the extracellular matrix (ECM), promoting breast cancer cell migration and invasion. LOX loss reduces tumor metastasis, while TGF-β and miR-200 enhance LOX expression, influencing matrix remodeling. Matrix stiffness alters CAF phenotype, boosting α-SMA expression and promoting proliferation and migration in response to PDGF. CAFs secrete MMPs (e.g., MMP-1, MMP-7, MMP-9), linked to tumor progression and poor prognosis. Tumor cells release TGF-β and TNF-α to stimulate MMP production by CAFs, aiding cancer cell invasion and protecting them from drugs like Taxotere. CAFs also exert mechanical pressure on tumor cells, promoting migration towards looser tissues.

### Suppressing immune cells

In a mouse breast cancer model, eliminating CAFs increased IL-2 and IL-7 levels, boosted dendritic and cytotoxic T cell numbers, and reduced tumor-promoting macrophages and regulatory T cells. In human breast cancer, CAFs recruited monocytes via MCP1, CXCL12, CCL2, and CCL16, leading to their differentiation into M2-like macrophages that exert immunosuppressive effects through the PD-1 axis. CAF-derived exosomes containing miRNAs inhibited T cell proliferation and promoted apoptosis via the miR-92/LATS2/YAP1/PD-L1 pathway.

**FIGURE 3:**
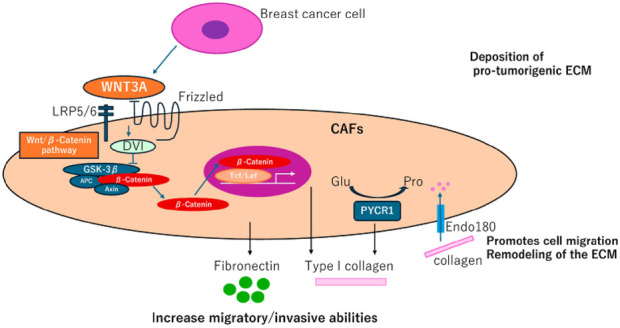
(CAFs) contribute to collagen production in breast cancer, presenting new therapeutic opportunities. Fibroblasts derived from adipocytes near breast tumor cells enhance the secretion of fibronectin and collagen I, facilitating tumor cell migration and invasion, often through activation of the Wnt/β-catenin pathway. Endo180 supports cell migration and extracellular matrix (ECM) remodeling by mediating collagen uptake. Additionally, PYCR1, a crucial enzyme in proline synthesis, plays a role in ECM production, promoting tumorigenesis and emerging as a potential therapeutic target.

Immunosuppression: In immunocompetent models, fibroblast activation protein alpha (FAP)-positive CAFs have been shown to drive immunosuppression and resistance to anti-PD-L1 immunotherapy, further complicating treatment outcomes (Stanton and Disis, 2016). Figure 3.

### Targeting CAFs to prevent cancer progression and improve therapy efficacy

Cancer-associated fibroblasts (CAFs) significantly influence cancer progression, promoting tumor growth in advanced stages through their plasticity and secretion of factors like TGFβ. Targeting CAFs involves strategies such as suppressing activation, reprogramming them to a quiescent state, reducing their presence in the tumor microenvironment, and inhibiting their tumor-promoting and immunosuppressive effects. Combining CAF-targeted approaches with other therapies may overcome resistance. Tumors often transform post-radiotherapy, becoming more sensitive to immunotherapy, making combination therapies promising. However, responses vary, emphasizing the need to understand CAF diversity and its impact on tumor signaling to develop effective treatments. Understanding the relationship between the diverse CAF secretome and tumor signaling could help identify effective combination treatment strategies (Stanton and Disis, 2016; Fiori et al., 2019).

### Therapeutic approaches targeting CAFs in breast cancer

Targeting cancer-associated fibroblasts (CAFs) in cancer therapy is difficult due to their diversity, but they are gaining attention as key targets for anti-tumor treatments. Understanding the specific CAF subsets linked to triple-negative breast cancer (TNBC) and their involvement in tumor progression is essential for creating effective treatments, especially immunotherapies. Investigating the interaction between CAFs, immune cells in the tumor microenvironment (TME), and proteins like biglycan could provide new therapeutic insights for treating breast cancer.

ER-antagonists like fulvestrant and methyl piperidino pyrazole reduce cell cycle and metabolism-related gene expression in CAFs, impacting tumor progression. Exploring the role of ER in CAFs within the tumor stroma could reveal new treatment possibilities for breast cancer.

In ER-negative breast cancers, 22% of stromal cells showed ER_36 positivity, indicating that studying its role in CAFs could reveal their contribution to the malignant phenotype in aggressive breast cancer (Fiori et al., 2019; Nagel et al., 2022).

Recent research emphasizes targeting cancer-associated fibroblasts (CAFs) in the tumor microenvironment (TME) to combat breast cancer growth, metastasis, and immunosuppression. Clinical trials are exploring therapies like hedgehog pathway inhibitors (sonidegib, vismodegib), TGF-β inhibitors (fresolimumab, M7824), and FAP-targeting approaches (vaccines, CAR-T cells). Agents such as ATRA, paricalcitol, and losartan aim to revert CAFs to a quiescent state, while galunisertib inhibits TGF-β signaling. Despite the promise of improved diagnosis, prognosis, and treatments with fewer side effects, CAF heterogeneity remains a significant challenge for clinical application.

Biglycan (BGN), encoded by the BGN gene, is primarily expressed in the stromal compartment of tumors and plays a role in the extracellular matrix, lymphangiogenesis, EMT, angiogenesis, and TGF-β signalling (Chen and Song, 2019).

CAF-targeting strategies aim to reprogram CAFs to a quiescent state using Vitamin A&D receptors, reduce CAFs in the TME through CAR-T therapy, vaccination, and monoclonal antibodies, and inhibit CAF-driven EMT, stemness, and metastasis. Additionally, targeting CAF-derived chemokines, cytokines, and exosomes can enhance T-cell access and improve treatment efficacy.

#### Targeting unique and highly expressed molecules in CAFs

FAP-based vaccines are being developed to treat tumors by targeting CAFs, showing promising results in breast cancer. These vaccines inhibit tumor growth, increase IFNγ and CD8-positive T cells, and enhance drug uptake by 70%. FAP-CAR T cells specifically target FAP-high CAFs, suppressing tumor growth. Additionally, FAP is used for drug delivery, such as conjugating epirubicin to FAP-specific peptides, which proved effective *in vitro* and *in vivo*. HDAC6 inhibition in CAFs reduces tumor growth, alters immune cell dynamics, and may serve as a potential therapeutic target for breast cancer (Broad et al., 2021).

#### Preventing CAF activation and reprogramming cafs to quiescent fibroblasts

Activation of resident fibroblasts plays a critical role in CAF generation and is a therapeutic target in breast cancer. In a TNBC model, Hedgehog-dependent CAF activation and ECM remodeling promoted CSC niches and docetaxel resistance, with the smoothened inhibitor sonidegib showing promise in preclinical studies and advancing to clinical trials. Losartan, an angiotensin-II receptor-1 inhibitor, reduces TGF-β, CCN2, and ET-1 signaling in CAFs, improving drug and oxygen delivery in tumors and inhibiting mammary tumor development in mice. Ongoing clinical trials are exploring combinations like camrelizumab, liposomal doxorubicin, and losartan (NCT05097248), and the use of all-trans retinoic acid (ATRA) to convert CAFs into quiescent fibroblasts (NCT04113863). Additionally, paricalcitol is being tested for its potential to inactivate CAFs in a phase I trial (NCT00637897) (Glabman et al., 2022).

#### Targeting Caf-secreted proteins and -associated signaling pathways

Cancer-associated fibroblasts (CAFs) influence tumor progression by secreting proteins like TGF-β, FGFs, and CXCLs, making their pathways valuable therapeutic targets. TGF-β inhibitors (e.g., fresolimumab, galunisertib) enhance T-cell activity, while dual-targeting M7824 shows strong antitumor effects. FGFR and CXCR inhibitors, such as erdafitinib and balixafortide, combat resistance and inhibit growth. Hyaluronan (HA) produced by CAFs is also associated with breast cancer malignancy (Naito et al., 2024).

PEGPH20, a hyaluronidase that degrades hyaluronan (HA), enhances drug efficacy by increasing tumor cell drug exposure. It improved the uptake of anti-PD-L1 antibody and lapatinib in animal models and reduced HA levels in clinical trials. However, a phase III trial in metastatic pancreatic cancer showed no benefit when PEGPH20 was combined with nab-paclitaxel/gemcitabine, and a phase II study in metastatic breast cancer was terminated due to enrollment challenges (NCT02753595). Fibronectin, produced by CAFs, promotes cancer progression, and immunization with its extra domain-A has been shown to reduce tumor growth and metastasis in mice. Inhibitors targeting the PIK3Cδ subunit of phosphatidylinositol-3-OH kinase (PIK3Cδ), such as CAL-101, have been effective in reducing tumor growth in breast cancer models (Glabman et al., 2022; Naito et al., 2024).

ANGPTL4(Angiopoietin-related 4 Protein), MMP13 (matrix metalloproteinase 13,collagenase 3) and STC-1(Stanniocalcina) were identified as STAT3-dependent mediators of CAF pro-tumorigenic functions. *In vitro* and *in vivo* studies demonstrated that inhibiting MMP13 impaired CAF activity, highlighting the potential of targeting STAT3-induced CAF-secreted proteins as a therapeutic strategy. This approach is clinically relevant, as a similar CAF-STAT3 signature is highly expressed in stromal cells of breast cancer patients, particularly those with basal-like disease, and is linked to shorter disease-specific survival (Chen et al., 2024). Table 2.

**TABLE 2:** Drug targeting CAF associated signaling in breast cancer investigated in clinical trials.

Target	Drug	Class	Mechanism	Trial ID and current status
Hedgehog	Vismodegib	Small-molecule inhibitor	Preventing CAF activation	NCT02694224 phase II. recruiting
	Sonidegib	Small-molecule inhibitor	Preventing CAF activation	NCT02027376, phase I, completed
Hyaluronic acid	PEGPH20	Hyaluronidase	Interfering with CAF-mediated desmoplasia	NCT02753595, phase II. terminated
Vitamin A metabolism	ATRA	Metabolite of vitamin A	Reprogramming CAFs	NCT04113863, phase 1. unknown
Vitamin D receptor	Paricalcitol	Small-molecule agonist	Reprogramming CAFs	NCT00637897, phase I, completed
Angiotensin receptor	Losartan	Small-molecule inhibitor	Reducing collagen and hyaluronan levels	NCT05097248 phase II, not yet recruiting
TGF-B	Fresolimumab	Monoclonal antibody	Neutralizing TGF-8	NCT01401062, phase II. completed
	Galunisertib	Small-molecule inhibitor	Preventing CAF activation and interfering with CAF-mediated signaling	NCT02672475, phase I, active, not recruiting
	M7824	Anti-PD LI/TGF-$ trap fusion protein	Preventing CAF activation and immune suppression, interfering with CAF-mediated signaling	NCT03524170 phase 1, active. not recruiting NCT04296942 phase I. completed NCT03579472, phase I, recruiting
FGFR	Erdafitinib	Small-molecule inhibitor	Interfering with CAF-mediated signaling	NCT03238196 Phase I, active, not recruiting
	AZD4547	Small-molecule inhibitor Small-molecule inhibitor	Interfering with CAF-mediated signaling Interfering with CAF-mediated signaling	NCT01202591, phase 1. completed NCT01791985, phase Ib/lla, completed
	Futibatinib	Small-molecule inhibitor	Interfering with CAF-mediated signaling	NCT04024436. phase II. recruiting
	Debio1347	Small-molecule inhibitor	Interfering with CAF-mediated signaling	NCT03344536 phase II. completed
CXCR4	Balixafortide	Small-molecule inhibitor	Interfering with CAF-mediated signaling	NCT01837095, phase I, completed

Abbreviations: CAF, cancer-associated fibroblast; PEGPH20, pegvorhyaluronidase alfa; ATRA, all-trans retinoic acid; TGF-ẞ, transforming growth factor beta; PD-LI, programmed cell death 1 ligand 1; FGFR, fibroblast growth factor receptor; CXCR4, C-X-C motif chemokine receptor 4.

## Discussion

Intratumor heterogeneity plays a crucial role in tumor evolution and is a leading cause of resistance to therapy. Tumors are now understood to be intricate systems comprising various cell types and surrounding components that interact dynamically (Chen et al., 2024; Bejarano et al., 2021).

As a key element of the tumor microenvironment (TME), Cancer-associated fibroblasts (CAFs), impact every stage of cancer progression, including initiation, growth, invasion, metastasis, and therapy resistance. Despite this, the mechanisms by which the TME, particularly CAFs, drives such heterogeneity are still not fully understood. This review highlights the research on CAFs, particularly in breast cancer, shedding light on their interactions with tumor cells and exploring current strategies to target CAFs for breast cancer treatment. Understanding the diversity of cancer-associated fibroblasts (CAFs) in breast cancer is crucial for developing effective therapeutic strategies.

While significant progress has been made in understanding their role, substantial challenges still exist in applying these findings to clinical treatments (Bejarano et al., 2021).

Cancer-associated fibroblasts (CAFs) play a key role in promoting cancer progression by interacting with immune and malignant cells, fostering tumor growth, metastasis, neoangiogenesis, extracellular matrix remodeling, and immunosuppression.

They also contribute to therapy resistance through the secretion of growth factors, cytokines, and matrix proteins. Triple-negative breast cancer (TNBC), an aggressive subtype accounting for 15%–25% of cases, lacks hormone and HER-2 receptors, making it unresponsive to targeted therapies. High CAF levels in TNBC are associated with tumor aggressiveness, recurrence, and poor outcomes.

Immunotherapy, particularly immune checkpoint inhibitors, offers a promising cancer treatment strategy by modulating the tumor microenvironment (TME) and immune cell activity to suppress tumor growth. However, these therapies benefit only a subset of triple-negative breast cancer (TNBC) patients, underscoring the need for further research to enhance their efficacy. In TNBC, cancer-associated fibroblasts (CAFs) play a significant role in immune suppression, promoting lipid-associated macrophages and facilitating immune evasion. Studies show substantial differences in gene expression between CAFs and normal fibroblasts, emphasizing the critical role of CAFs in tumor progression and the potential for targeting them to improve TNBC outcomes. Studies have explored the association between CAF subtypes, tumor purity, immune cell infiltration, and the effectiveness of immune checkpoint blockade (ICB). The findings indicate that CAFs contribute to a poor prognosis by aiding tumor cells and inhibiting immune responses, particularly by suppressing CD8^+^ T cells. As tumor-infiltrating T cells are critical predictors of ICB efficacy, TNBC outcomes remain challenging, prompting efforts to discover new therapeutic targets and enhance the use of existing treatments. A deeper understanding of chemotherapy resistance mechanisms is vital to improving strategies for this aggressive cancer subtype (Mehraj et al., 2021; Emon et al., 2018). However, to the best of our knowledge, no research has yet investigated whether CAFs from different breast cancer subtypes exhibit subtype-specific gene expression patterns. Specifically, it has been demonstrated that the gene expression profile of CAFs from Her2+ breast cancers differs significantly from that of CAFs derived from ER + or TNBC breast cancers. Fibroblast heterogeneity has been observed in various organs, including the lung, skin, sclera, and orbit. Additionally, Sugimoto and colleagues showed that the expression of different fibroblast markers is heterogeneous within the tumor stroma in mouse models of breast and pancreatic cancers, using immunohistochemical analyses. While several studies have created gene expression profiles for breast cancer-associated fibroblasts, none have classified their results according to tumor subtypes (Allinen et al., 2004; Miki et al., 2020).

Studies have shown that CAF-related patterns are closely linked to T-cell differentiation, which influences TNBC prognosis. Higher infiltration of activated CD4^+^, CD8^+^, and gamma delta T cells is associated with better outcomes, as these cells inhibit TNBC progression. CAFs have been identified as potential predictors of clinical outcomes and immunotherapy responsiveness in TNBC patients. Research by Allinen et al. and Singer et al. highlighted significant differences in gene expression profiles between CAFs and normal fibroblasts, with CAFs exhibiting increased expression of tumor-promoting genes (Allinen et al., 2004).

Bauer et al. analyzed gene expression profiles of fibroblasts from six matched breast cancer and adjacent normal tissues, revealing significant differences in genes associated with paracrine signaling, transcriptional regulation, extracellular matrix, and cell adhesion/migration. However, these studies were not designed to explore subtype-specific CAF differences due to small sample sizes and underrepresentation of less common subtypes like Her2+ and TNBC, limiting insights into these categories (Emon et al., 2018).

Recent studies have shown that CAFs play a role in angiogenesis, tumor cell proliferation, treatment resistance, immunomodulation, and metastasis in solid tumors like breast cancer. However, current research on the role of CAFs in breast cancer is still limited. According to several study, the presence of CAFs in the tumor microenvironment (TME) is higher in patients with a worse prognosis, suggesting that CAFs could be an independent prognostic factor.

With advancements like single-cell sequencing and novel *in vitro* models, research on CAFs is advancing quickly. Efforts to develop CAF-targeting therapies are underway, providing new avenues for combating cancer. Given their involvement in angiogenesis, metastatic invasion, and maintaining stemness, CAFs have become a key focus as potential therapeutic targets in breast cancer.

## Conclusion and perspectives

In summary, Cancer-Associated Fibroblasts (CAFs) play a crucial role in the development and progression of breast cancer. Recent research has highlighted the diverse origins and functions of CAFs within the tumor microenvironment, showing that they can either promote or suppress tumor growth depending on various factors. The heterogeneity of CAFs contributes to their involvement in tumor-promoting mechanisms such as stemness, metastasis, migration, invasion, angiogenesis, and therapy resistance, through processes like extracellular matrix (ECM) remodeling, immune modulation, and altered metabolism.

Studies using advanced techniques like single-cell RNA sequencing (scRNA-seq) have identified unique CAF subtypes and potential markers, providing insights into their molecular and phenotypical differences. This deeper understanding could help address issues related to therapy resistance and improve cancer cell targeting. CAF-induced resistance to therapy is a significant factor in tumor progression and reduced patient survival. However, the lack of exclusive markers, such as αSMA or FAP, on CAFs complicates the development of targeted treatments. Gaining more knowledge about how CAFs contribute to cancer development in response to various therapies could lead to better treatment strategies and improved therapeutic outcomes.

Recent research suggests that cancer-associated fibroblasts (CAFs) could be valuable targets for therapy in triple-negative breast cancer (TNBC). These fibroblasts significantly shape the tumor microenvironment by promoting tumor growth, metastasis, neoangiogenesis, extracellular matrix remodeling, and immunosuppression. Through the secretion of growth factors, inflammatory cytokines, and matrix proteins, CAFs drive tumor progression and contribute to treatment resistance. Consequently, a deeper understanding of the heterogeneity of CAFs in breast cancer is crucial for developing effective therapeutic strategies.
